# Regret, policy, and discontinuance of gas vehicles: a cross-national study of Malaysia and Thailand

**DOI:** 10.1038/s41598-025-07259-0

**Published:** 2025-07-02

**Authors:** Wanamina Bostan Ali, Long Kim, Gulmira Issayeva

**Affiliations:** 1 Faculty of Management Sciences, Prince of Songkla University, Songkla, Thailand; 2 Center of Excellence in Logistics and Business Analytics (LOGBIZ), School of Accountancy and Finance, Walailak University, Nakhorn Si Thammarat, Thailand; 3 Candidate of Economic Sciences, Department of Finance, M.Auezov South Kazakhstan University, Shymkent, Kazakhstan

**Keywords:** Regret, Satisfaction, Attitude, Discontinued intention, Carbon dioxide, Climate-change adaptation, Psychology and behaviour, Sustainability

## Abstract

As a psychological element, regret functions as a reflective emotion that motivates people to assess their past behaviors along with their decision-making processes and life paths. While previous research has shown that regret determines satisfaction and attitudes and influences discontinued intentions, there is a lack of empirical data regarding its effect on travelers’ decisions to stop using gas vehicles. This research explores how regret affects satisfaction and attitudes toward discontinued intentions in Malaysia’s and Thailand’s gas vehicle industries and examines government policy as a moderating factor. The research team invited 700 participants split equally between Malaysia and Thailand to complete a structured questionnaire. The path analysis of data demonstrated that regret had a substantial impact on traveler satisfaction across both countries. The study found that traveler attitudes in both national contexts were affected by both regret and satisfaction. Travelers in Malaysia and Thailand discontinued their future visits based on their levels of regret and satisfaction, which also shaped their attitudes. The study showed that government policy served as a moderating factor between regret and discontinued intention in both countries, which emphasizes how regulations affect consumer behavior. The study results reveal important details about how psychological aspects combine with policy responses to drive behavioral patterns related to gas vehicle use.

**Supplementary Information:**

The online version contains supplementary material available at 10.1038/s41598-025-07259-0.

## Introduction

The profound influence of technology on users’ tasks and activities is evident in its capacity to enhance convenience, reduce costs, improve work efficiency, and boost productivity^1^. Nevertheless, alongside these advantages, technology also introduces challenges (e.g., frustration, stress, emotional and physical exhaustion, and unforeseen risks) that collectively contribute to negative user experiences^2^. Meanwhile, research found that the adverse effects of technology can also cause societal concerns such as: mental health impact (56%), diminished work productivity due to distractions (73%), and physical health issues (37.7%)^3,4^. When the consequences of technology outweigh its benefits, users may experience regret and guilt regarding their consumption patterns, a phenomenon that resonates at both individual and societal levels^5^. From a consumer psychology perspective, this regret can significantly diminish satisfaction because individuals who feel dissatisfied with a product no longer derive enjoyment from its use, resulting in reduced overall contentment^6^. Besides, regret influences user attitudes by fostering feelings of guilt and stress, which culminate in unfavorable perceptions of the product^7^. Furthermore, regret has been identified as a critical factor linked to discontinued usage intentions. Especially, when users recognize or experience the negative repercussions of a product, they are more likely to cease its use^8^. Thus, regret emerges as a critical psychological determinant that affects consumer satisfaction and attitudes and drives decisions to discontinue technology usage.

The most significant negative consequence of technological use manifests as environmental damage, including ecological and climate change, which are now key global concerns^9,10^. Gas vehicles, including trucks, cars, and motorbikes, consume large quantities of various fuels such as diesel, E85 flex fuel, compressed natural gas, and liquefied petroleum gas^12^. These vehicles emit carbon dioxide (CO₂) into the atmosphere. Travelers across the globe received warnings regarding the environmental dangers posed by their gasoline-powered vehicles, which could affect surrounding ecosystems^11^. These chemical substances impact both the quality of the air and the overall health of the environment. Travelers who understand the environmental impacts of using gas vehicles experience serious anxiety about the planet’s future. Travelers experience feelings of guilt because they recognize their responsibility in damaging both their immediate environment and the broader global environment through their use of current technology. The gas vehicle industry faces long-term threats to consumer retention and business viability because intensifying consumer regret will drive customers to discontinue product use with these companies^8^. Therefore, the vehicle industry requires thorough research that establishes the effects of traveler regret on gas vehicles.

Research shows that consumer regret functions as a psychological element that affects satisfaction^5^, attitude^7^, and discontinued intention^13^. The changes in discontinued intention were driven by satisfaction^14^ and attitudes^15^. A government policy that restricts harmful products that affect behavioral decisions^16^ can make users aware of regrettable product choices while prompting them to discontinue usage. The number of studies examining human regret as a psychological element that forms the basis for travelers’ decisions to stop using gas is limited despite various researchers claiming its importance. This situation leads to a research question “What if users regret using the gas vehicles, is discontinuance inevitable?”. So far, this question has not been answered or defined in current research literature. Consequently, the vehicle industry’s policymakers, product engineers, and entrepreneurs experience significant uncertainty without clear answers to this research question because they hesitate to develop environmentally friendly vehicle alternatives. Hence, this research introduces a new model to explore how feelings of regret influence satisfaction and attitudes toward the intention to discontinue certain practices, with government policy serving as a moderating factor. To guide the research process, the following sub-research objectives are explored:

1. To examine the impacts of regret on traveler satisfaction.
2. To examine the impacts of regret and satisfaction on traveler attitude.
3. To examine the impacts of regret, satisfaction, and attitude on discontinued intention.
4. To examine the moderating impact of government policy on the link between regret and discontinued intention.

This research endeavor will enable academic learners to develop an intricate comprehension of how psychological elements and governmental regulations influence users’ decisions to stop using gas-powered vehicles. The research findings may clarify these industry dynamics to provide vehicle manufacturers with essential insights for understanding consumer needs and guiding investment toward alternative technologies that benefit users while reducing ecological impacts. Advancements that integrate cutting-edge technologies with sustainable environmental practices can improve client retention by meeting modern consumer demands.

## **Literature review**

### Discontinue intention and research gap analysis

The study of consumer behavior has garnered significant attention among marketing scholars, as it enables firms to identify strategies for meeting customer needs and enhancing sales performance^17^. Within this domain, discontinued intention has emerged as a focal point for researchers that provides implications for consumer decisions to cease using specific products or switch to alternative brands in the future^18^. Maier et al.^19^ define discontinued intention as an individual’s propensity to discontinue engagement with a firm’s services, whether temporarily or permanently, at a subsequent point in time. Factors contributing to such intentions are often linked to diminishing product popularity and a perceived decline in the benefits associated with the product. These can significantly influence consumer loyalty and retention^18^. Understanding these dynamics is essential for firms seeking to mitigate attrition and sustain competitive advantage in increasingly dynamic markets.

The theory of planned behavior suggests that individuals develop intentions to perform an action once their attitudes and subjective norms transform. A person’s intention produces actual performance, which logically occurs between individuals within this context. The decision to stop using any services from the current provider emerges when any consumer forms an intention to discontinue those services. Pang et al.^20^ assert that consumers demonstrate their intent to discontinue services by requesting service cancellation when their intention to quit reaches high levels. The incident will result in major impacts that deeply affect both companies’ financial results and their operational activities within this area. Because discontinuation issues represent a critical challenge for businesses, they must perform thorough research to detect root causes that drive consumers to end their service usage. The existing body of literature presents multiple investigations that address this particular issue.

Various research groups have identified and analyzed a multitude of factors influencing discontinued intention within the vehicle industry, contributing to a complex understanding of consumer behavior. Hardman and Tal^21^ examined the impact of satisfaction, price, safety, reliability, convenience, and refueling costs on the propensity to discontinue usage, establishing a foundational framework for subsequent investigations. Building on this foundation, Khan et al.^22^ extended the inquiry by evaluating the influence of driving range performance, future viability, safety concerns, and dissatisfaction levels, thereby highlighting the interplay between technological advancements and consumer perceptions. Lu and Shi^23^ further enriched the discourse by exploring the roles of perceived risk, attitude, subjective norm, and perceived behavioral control, emphasizing the psychological and social dimensions of decision-making. Then, Dua et al.^24^ examined the effects of recharge times, charge unavailability, price replacement costs, and perceived value, shedding light on the practical challenges associated with adopting alternative technologies. Complementing these findings, another study by Lu and Shi^25^ incorporated switching costs and perceived value into their analysis, alongside previously studied constructs such as perceived risk, subjective norm, and perceived behavioral control, thereby offering a comprehensive perspective that integrates economic, psychological, and technological considerations.

**Fig. 1:**
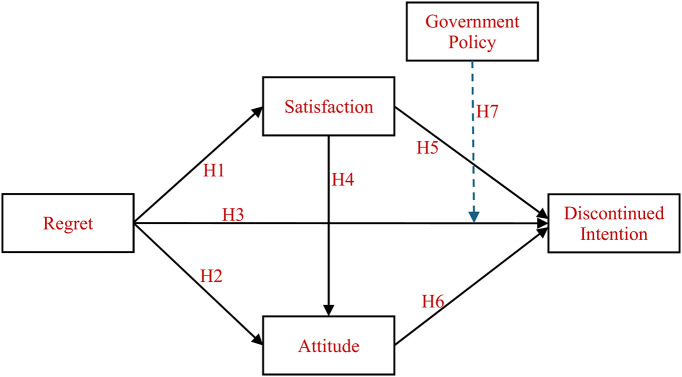
Research model of discontinued intention.

Analysis of research gaps indicates that regret influences satisfaction^5^, attitude^7^, and discontinued intention^13^, but researchers also identify satisfaction^14^ and attitudes^15^ as predictors of discontinued intention. The government policy, which affects individuals’ behaviors and decision-making processes^16^, also shapes consumer discontinued intention. The existing literature lacks extensive examination of how these factors combined can explain the development of discontinued intention in the gas vehicle industry. The existing literature lacks both sufficient information and empirical evidence that would explain current findings. This study seeks to bridge the existing research gap by investigating how regret affects satisfaction levels along with attitudes and discontinued intention. This research distinguishes itself by examining how government policy influences the relationship between regret and discontinued intention. The moderating testing demonstrates the significant relationship between regret as a psychological element and discontinued intention, which represents the choice to stop product usage. Figure 1 displays the novel research model that the study introduces.

### Regret

Regret refers to an emotion that a person experiences after realizing that his or her situation would be better if only the person had decided differently^5^. Regret is considered a consequential emotion that negatively influences individuals’ well-being and decision-making processes. To illustrate, people who feel satisfied also seem worried about past behaviors that they now regret^26^. Consumers experiencing regret frequently recall their previous service consumption experiences that resulted in negative outcomes. Such a condition increases customers’ worries, which then generate high frustration and stress that impact their service consumption experience. Hence, the presence of a strong tendency to experience regret reduces customer satisfaction with businesses^5^.

Regret functions as a psychological element that affects emotions and reshapes people’s attitudes toward both previous and present product experiences by altering their beliefs and perceptions^7^. Consumers who feel regret over a specific product use typically experience both guilt and dissatisfaction with their choice. Their emotional reaction to regretful experiences causes a reassessment of their past product usage while questioning the actual benefits promised by the product. The emergence of skepticism leads people to distrust products and develop adverse attitudes because they start seeing them as potential risks. This perceptual shift creates a higher chance of avoidance behaviors because consumers want to prevent additional contact with expected risks^27^.

Regardless of its relationship with discontinued intention, when a person regrets doing something, he or she often recognizes the negative outcome of his or her past choice^13^. This psychology triggers his or her own fear and alerts the person to avoid repeating harmful behaviors or patterns that can lead to consequences. Furthermore, it has been found that when regret arises, guilt and disappointment start increasing significantly. At this rate, it can drive the person to halt his or her behavior, attitude, and decision. Therefore, the rise of regret significantly promotes the intensity of discontinued intention^8^. Hence, the hypotheses are proposed below:

#### H1

Regret negatively influences traveler satisfaction.

#### H2

Regret negatively influences traveler attitude.

#### H3

Regret positive influences discontinued intention.

### Satisfaction

The achievement of customer satisfaction is determined by comparing the customer’s expectations of the desired product or service with their actual experience after purchase^28^. The evaluation of customer satisfaction includes two fundamental dimensions, which are cognitive and emotional^29^. The cognitive dimension consists of logical and rational assessments in purchasing products or services, while the emotional dimension reveals customers’ feelings, such as joy or pleasure, to confirm their expectations.

Customers who are content with their product consumption find that the performance aligns with their expectations due to its association with their attitudes^30^. Customers frequently use the product and maintain their trust and confidence in the company. The positive experience leads customers to develop a more optimistic outlook toward the firm. Customers’ positive feelings about a firm persist after receiving satisfying service experiences from it. When customer satisfaction rises, it generates higher customer attitudes toward the firms^31^.

Customer appreciation through satisfaction shows how their responses to product and service offerings relate to discontinued intention^14^. Customer appreciation allows them to maintain confidence in using additional services and products from the companies. The customer’s loss of confidence in service or product offerings is triggered by their dissatisfaction with them. As a result, customers develop an increased likelihood of discontinuing service or product use with these firms^32^. Hence, the following hypotheses are proposed:

#### H4

Satisfaction positively influences traveler attitude.

#### H5

Satisfaction negatively influences a traveler’s intention to discontinue gas vehicles.

### Attitude

A consumer attitude represents a mental state that combines beliefs and emotions, which drive people to take action toward buying services or products^31^. By understanding consumer attitudes, we can identify decision-making information and behavior patterns regarding brand selection among firms.

People decide whether to keep or stop using particular services based on how their attitude measures against discontinued intention^15^. Individuals who possess positive or high attitudes towards existing service firms will likely continue using them in the future based on behavioral intentions. A low attitude towards service firms results in a reduced likelihood of continued use^33^. Thus, the following hypothesis is proposed:

#### H6

Attitude negatively influences a traveler’s intention to discontinue gas vehicles.

### Government policy on gas vehicles

The government policy represents an official statement made by a country’s government that guides its political activities and plans related to a particular issue^34^. Government policy determines both how individuals make decisions and the overall direction of their lives^35^. Research has demonstrated that modifications in government policy significantly affect how different elements influence consumer decisions and behaviors towards businesses^16^. Government policies direct individual behavior through several mechanisms, which include incentives and regulations as well as awareness campaigns^36,37^. When government policy becomes involved, individual users experience regret about side effects of a certain product, which then increases their desire to stop using the product. Through decision-making process modifications and regret behavior mitigation mechanisms, government policy amplifies the link between regret and discontinuation intentions.

Particularly, the Malaysian government has warned about the effects of carbon dioxide (CO₂) emissions from gas vehicles on air quality and environmental conditions. Subsequently, this policy motivates travelers to use bioethanol- and biodiesel-powered vehicles as alternatives to hydrocarbon fuels to achieve environmental sustainability and carbon neutrality^38^. Similarly, the Thai government urges travelers to transition to transportation options that emit lower levels of carbon dioxide (CO₂) or utilize renewable energy sources to achieve better environmental sustainability^10^. Ground transportation between Malaysia and Thailand serves as the primary support system for tourist destinations up to this point^39^. Government policy involvement educates gas vehicle travelers about how carbon dioxide (CO₂) impacts both individual health and environmental conditions^40^. This situation triggers feelings of guilt in travelers about their past decisions to use gas vehicles, which contributed to environmental damage. A growing number of travelers between Malaysia and Thailand have adopted a new trend of environment protection. This emphasizes environmental awareness and leads them to abandon the use of gas vehicles^41^. Therefore, the following hypothesis is proposed:

#### H7

Government policy moderates a link between regret and discontinued intention.

## Methods

### Sample characteristics and size

The research took place within the context of Malaysia and Thailand. The research team contacted 700 participants, evenly divided between Malaysia and Thailand, to participate in the study using survey questionnaires. The research used sample characteristics to study the reasons why travelers in both countries stopped using gas vehicles. The researchers selected current gas vehicle users as their target respondents because they were best suited to provide the necessary answers for the research objective. Each country’s respondents consisted of various traveler categories, including campers, business travelers, adventurers, tourists, and visitors who used gas vehicles to reach their destinations. The study did not include participants who traveled with electric vehicles because they did not fit the research objectives.

The selection of an appropriate sample size was guided by calculations based on either known or unknown population parameters, ensuring alignment with the quality benchmarks for generalizability proposed by Kim et al.^42^. According to the sample size and quality check outlined in Table 1, sample sizes were categorized into distinct quality levels, ranging from “10 = Very Poor Quality” to “1,000 = Excellent Quality.” To achieve a balance between practicality and robustness, this study targeted a minimum of 350 data sets from each country—Malaysia and Thailand—positioning the sample within the “Good” to “Very Good” quality range. By collecting 350 responses per country, the total dataset encompassed 700 observations, thereby enhancing the reliability and generalizability of the findings across both national contexts.

**Table 1:** Sample size and quality classification.

Sample size	Quality
10	Very poor
50	Poor
200	Fair
300	Good
500	Very good
1,000	Excellent

Following the recommendation of Kim et al.^42^.

### Data collection

Researchers submitted the research proposal and survey instrument to the Ethics Committee in Human Research at Walailak University for review. Under ethical compliance, the proposed project, along with the informed consent process, successfully passed the review and received approval from the Ethics Committee in Human Research at Walailak University. This approval was granted in alignment with the principles outlined in the Declaration of Helsinki, under the approval number WUEC-23-342-01. Following this formal approval, researchers proceeded to the subsequent stages of the study, including pilot testing and full-scale data collection, which are elaborated upon in the following sections.

Before proceeding with full-scale data collection, this study incorporated a pilot testing phase designed to evaluate the reliability and clarity of the survey measurements. The pilot test involved 30 participants from each country, Malaysia and Thailand, whose responses were analyzed to assess content reliability using Cronbach’s Alpha (with a threshold of > 0.7). As detailed in the Appendix section, all variables across both countries achieved Cronbach’s Alpha scores exceeding 0.7, confirming sufficient content reliability. Based on the feedback received during the pilot testing, researchers further refined the questionnaire by modifying and revising specific items to enhance content clarity and ensure the instrument’s effectiveness. This iterative process aimed to address potential ambiguities and improve the overall quality of the survey before its deployment in the main study.

Researchers proceeded to gather comprehensive data from travelers across Malaysia and Thailand. Researchers started by using convenience sampling to randomly approach travelers at easily accessible locations, including public streets, parks, and the Malaysian-Thailand border areas. Researchers obtained their data collection diversity by adhering to two key practical aspects. Each group or family had only one representative selected by the researchers for participation. By using this approach, researchers can decrease the amount of redundant survey responses and thereby substantially minimize potential biases. Researchers alternated their survey locations across different weeks to gather data from various respondents. As a result, researchers minimized the possibility of encountering the same respondents across multiple survey locations and times.

During the survey operations, respondents were required to answer a screening question: “Have you traveled by gas vehicles to any tourist destination in this country?” This ensured that only participants meeting the study’s minimum eligibility criteria were included. Upon passing the screening question, respondents were asked to provide their consent before proceeding. Once consent was granted, participants were presented with a set of questionnaires, which took approximately 10 to 15 min to complete. To enhance data richness and participant recruitment, researchers employed a snowball sampling method at the end of the survey, optionally requesting respondents to suggest other potential participants who might meet the study’s criteria. However, this step was entirely voluntary for the respondents. Following three months of data collection, conducted between August and October 2024, researchers successfully gathered 350 valid responses from each country, Malaysia and Thailand, ensuring a robust dataset for subsequent analysis.

### Survey measurement

This study incorporated a survey measurement designed to examine five primary variables: regret, satisfaction, attitude, discontinued intention, and government policy. The items for each variable were derived from established prior literature, ensuring their relevance and theoretical grounding. Specifically, the items measuring regret were adapted from Nawaz et al.^43^, while those assessing satisfaction were drawn from Amin et al.^44^. Additionally, the construct of attitude was operationalized using items adapted from Smith^45^, and the variable of discontinued intention was based on the work of Maier et al.^19^. Finally, the items related to government policy were adapted from Sukma et al.^46^. To ensure contextual relevance, all items were carefully revised and modified to align with the experiences and perspectives of travelers who utilized gas vehicles. The survey employed a 5-point Likert scale, ranging from 1 (strongly disagree) to 5 (strongly agree), to capture respondents’ opinions effectively. The complete survey instrument, including all adapted and modified items, is detailed in the Appendix section for transparency and reference^47^.

### Common method of Bias

Researchers used questionnaires to collect data, and thus all responses reflected personal opinions of the respondents. These answers emerged from self-reported data, which necessitated the use of Common Method of Bias (CMB) analysis to evaluate potential biases in the existing information. When Harman’s single-factor testing shows a random factor with variance scores above 50%, this reveals potential bias in the collected data. SPPS software enabled CMB statistics by extracting one factor without rotation based on component analysis principles. The variance scores obtained for a randomized factor in Table 2 were 36.509% below 50%, which indicated this research’s data collection process was not biased.

**Table 2:** Common method bias statistics.

Total variance explained						
Component	Initial eigenvalues			Extraction sums of squared loadings		
	Total	% of Variance	Cumulative %	Total	% of Variance	Cumulative %
1	12.603	36.509	36.509	12.603	36.509	36.509
2	3.722	11.125	39.201			
3	2.609	7.902	42.336			
4	1.117	4.025	44.023			
5	0.909	3.011	47.008			
6	0.836	2.856	49.201			
7	0.752	1.905	51.360			
8	0.701	1.136	53.031			
9	0.697	0.988	55.825			

Extraction Method: Principal Component Analysis.

### Data normality and Multi-collinearity statistics

The research provided comprehensive statistical tests to examine data normality and established multicollinearity statistics. The research required skewness and kurtosis scores to be within the + 1.96 to -1.96 range to achieve data normality^48^. Multi-collinearity statistics showed the presence of multi-collinearity when variance inflation factor (VIF) exceeded 10, which led to potential conflicting regression results among the variables^49^. The research gathered sufficient data normality statistics from Table 3 because all variables achieved skewness and kurtosis scores that met the required thresholds. The VIF scores remained under 10, which confirmed that multicollinearity was not a significant problem in this research.

**Table 3:** Data normality and Multi-collinearity Statistics.

Variable	Data Normality		Collinearity Statistics
	Kurtosis	Skewness	VIF
Regret	0.427	1.205	5.208
Satisfaction	1.229	0.622	4.336
Attitude	0.791	0.588	3.287
Government Policy	0.922	0.479	4.900
Discontinued Intention	0.860	0.501	1.208

## Results

### Model measurement and model fit

The researchers utilized Amos Software (Version 24) to perform a path analysis technique for data examination. The analysis technique included two primary statistics, which consisted of model measurement and model fit, according to statistics guidelines from reference^50^. The following section displays the model measurement and model fit statistics for Malaysia and Thailand.

In the assessment of model measurement, the reliability of the model was initially examined using Cronbach’s Alpha (α) and Composite Reliability (CR), with scores required to exceed 0.7 to indicate acceptable reliability. As presented in Table 4, all variables achieved sufficient reliability statistics, confirming the model’s reliability for both countries. Subsequently, the analysis extended to evaluating convergent and discriminant validity. For convergent validity, the average variance extracted (AVE) for each variable was assessed, with a minimum threshold of 0.5 required to demonstrate adequate explanatory power. Discriminant validity was confirmed when the correlation coefficients between variables were lower than the square root of the AVE scores, ensuring that variables were sufficiently distinct from one another. The results reported in Table 4 indicate that the research model satisfied both convergent and discriminant validity criteria for both countries, as all relevant statistical measures exceeded the established thresholds, thereby affirming the robustness of the measurement model.

In model fit, all critical fitness indicators representing model fit of Malaysia and Thailand were evaluated as follows. First, chi-square index/ degree of freedom (CMIN2/df) had to be equal to or below 3. Second, Goodness of Fit Index (GFI), Normed Fit Index (NFI), and Comparative Fit Index (CFI) had to obtain scores equal to or above 0.9. Third, Adjusted Goodness of Fit Index (AGFI) had to obtain scores equal to or above 0.8. Fourth, Root Mean Square Error of Approximation (RMSEA) had to obtain scores equal to or below 0.08. Finally, p-value for Close Fit (PCLOSE) had to obtain scores equal to or above 0.05. In Table 5, the fitness statistics extracted from the Amos Software revealed that each fitness indicator in both Malaysia and Thailand passed the minimum requirements; hence, the research models of both countries were fit enough to perform the path analysis technique.

**Table 4:** Model measurement Statistics.

Variable	Thailand								Malaysia							
	(α)	CR	AVE	1	2	3	4	5	(α)	CR	AVE	1	2	3	4	5
Regret	0.88	0.93	0.71	**0.87**	-0.44	-0.38	0.51	0.47	0.89	0.73	0.64	**0.82**	-0.49	-0.50	0.33	0.31
Satisfaction	0.79	0.84	0.70		**0.93**	0.50	-0.41	-0.55	0.74	0.75	0.69		**0.90**	0.39	-0.55	-0.54
Attitude	0.90	0.86	0.68			**0.84**	-0.33	-0.49	0.75	0.79	0.72			**0.81**	-0.58	-0.60
Discontinued Intention	0.91	0.75	0.66				**0.89**	0.58	0.81	0.88	0.78				**0.95**	0.53
Government Policy	0.82	0.77	0.78					**0.79**	0.84	0.90	0.70					**0.88**

Note: the bolded numbers indicate the square root scores of AVEs.

**Table 5:** Model fit Statistics.

Indicators	Malaysia	Thailand	Thresholds	Results
CMIN^2^/df	2.570	2.208	≤ 3	Good
GFI	0.958	0.987	≥ 0.9	Good
NFI	0.984	0.988	≥ 0.9	Good
CFI	0.966	0.994	≥ 0.9	Good
AGFI	0.980	0.990	≥ 0.8	Good
RMSEA	0.031	0.063	≤ 0.08	Good
PCLOSE	0.087	0.109	≥ 0.05	Good

**Fig. 2:**
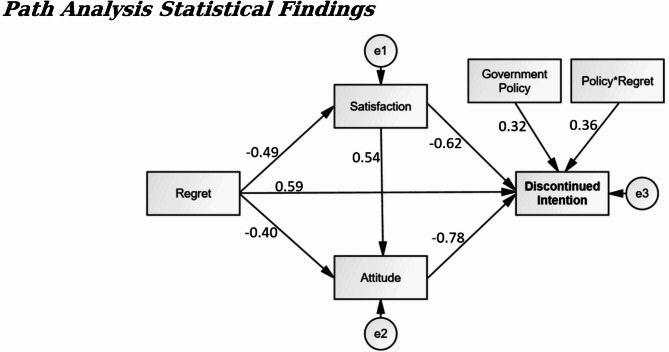
Path analysis statistics for Thailand.

**Fig. 3:**
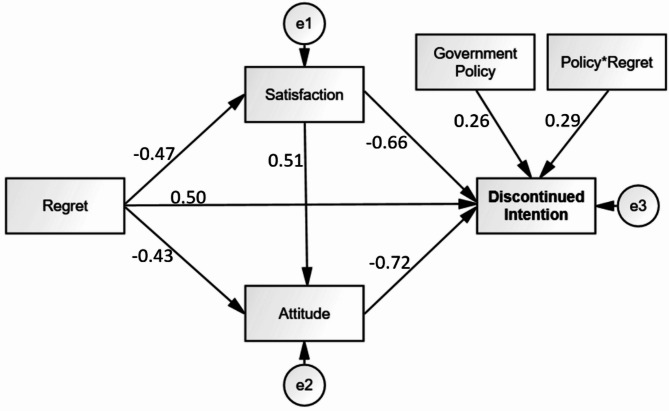
Path analysis statistics for Malaysia.

The key statistical results were shown in Figs. 2 and 3, and Table 6, which included two main types of analysis: direct path statistics and moderating path statistics for both Thailand and Malaysia. In the direct path analysis, regret had a strong negative effect on satisfaction, with standardized coefficients of β = -0.49 (*p* < 0.05) for Thailand and β = -0.47 (*p* < 0.05) for Malaysia. Similarly, attitude was significantly shaped by both regret and satisfaction; specifically, regret exhibited a negative influence on attitude, with β = -0.40 (*p* < 0.05) for Thailand and β = -0.43 (*p* < 0.05) for Malaysia, while satisfaction positively impacted attitude, yielding β = 0.54 (*p* < 0.05) for Thailand and β = 0.51 (*p* < 0.05) for Malaysia. Furthermore, discontinued intention was significantly influenced by multiple factors: regret displayed a positive impact, with β = 0.59 (*p* < 0.05) for Thailand and β = 0.50 (*p* < 0.05) for Malaysia; satisfaction exerted a negative influence, with β = -0.62 (*p* < 0.05) for Thailand and β = -0.66 (*p* < 0.05) for Malaysia; and attitude also negatively affected discontinued intention, with β = -0.78 (*p* < 0.05) for Thailand and β = -0.72 (*p* < 0.05) for Malaysia.

**Fig. 4:**
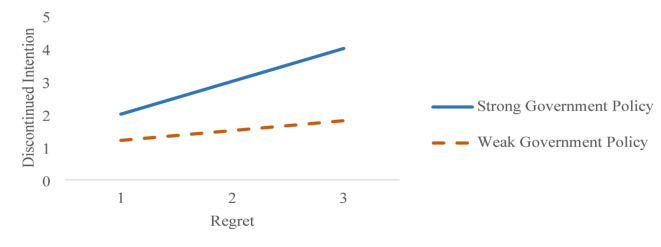
Moderating testing diagnostics for Thailand.

**Fig. 5:**
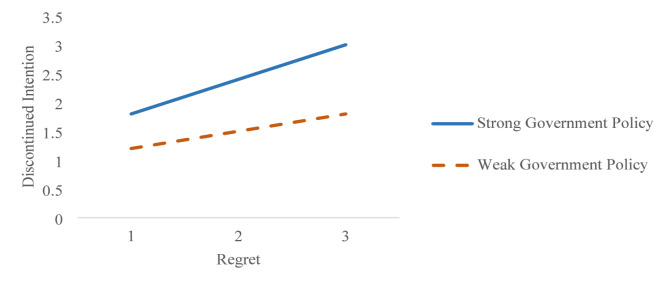
Moderating testing diagnostics for Malaysia.

The moderating path statistics revealed that discontinued intention was significantly influenced by the moderating variable “government policy” and the interacting variable “policy regret.” Specifically, government policy demonstrated a positive influence on discontinued intention, with standardized coefficients of β = 0.32 (*p* < 0.05) for Thailand and β = 0.26 (*p* < 0.05) for Malaysia. Similarly, the interaction term “policy regret” also exhibited a significant impact, yielding β = 0.36 (*p* < 0.05) for Thailand and β = 0.29 (*p* < 0.05) for Malaysia. Figure 4, representing the moderating diagnostics for Thailand, illustrated that as government policy shifted from weak to strong, discontinued intention increased from 1.5 to 4, while regret remained constant. Likewise, Fig. 5, depicting the moderating diagnostics for Malaysia, showed that discontinued intention rose from 1.6 to 3 under the same conditions of increasing government policy strength, with regret held constant. These findings underline the critical role of government policy in shaping the relationship between regret and discontinued intention among travelers in both Thailand and Malaysia, highlighting its capacity to amplify or mitigate the effects of regret on consumers’ decisions to discontinue usage.

**Table 6:** Critical statistics and hypotheses summary.

Chanel A: direct path statistics							
Independent variable	Dependent variable	St.D Beta (β)		*p*-value		Hypotheses result	
		Thailand	Malaysia	Thailand	Malaysia	Thailand	Malaysia
Regret	Satisfaction	-0.49	-0.47	0.000*	0.000*	Supported	Supported
Regret	Attitude	-0.40	-0.43	0.000*	0.000*	Supported	Supported
Satisfaction		0.54	0.51	0.000*	0.000*	Supported	Supported
Regret	Discontinued Intention	0.59	0.50	0.000*	0.000*	Supported	Supported
Satisfaction		-0.62	-0.66	0.000*	0.000*	Supported	Supported
Attitude		-0.78	-0.72	0.000*	0.000*	Supported	Supported

Chanel B: Moderating path statistics							
Moderating & interacting variables	Dependent variable	St.D Beta (β)		p-value		Hypotheses result	
		Thailand	Malaysia	Thailand	Malaysia	Thailand	Malaysia
Government Policy	Discontinued Intention	0.32	0.26	0.000*	0.012*	Confirmed	Confirmed
Policy*Regret		0.36	0.29	0.000*	0.008*	Supported	Supported

Note: Standard statistical error is 5%.

## Discussion

Based on the impact on satisfaction, regret negatively influenced traveler satisfaction in Malaysia and Thailand confirmed hypothesis 1. Previous research^5,26^ has established a strong relationship between regret and satisfaction, which aligns with the findings presented here. The study discovered that regret impacted Thai travelers’ satisfaction more than Malaysian travelers’ satisfaction, even though the effect remained consistent across both countries. The small variance between Thailand and Malaysia shows cultural guilt norms that influence Thai travelers through societal expectations and values more than Malaysian travelers, even when considering environmental impacts of gas vehicle usage. The recognition of their gas vehicle consumption behavior made Thai travelers feel more guilty compared to Malaysian travelers and caused them to experience higher levels of dissatisfaction with their consumption actions. Travelers showed frustration and disappointment when gas vehicles failed to meet their expectations for both travel benefits and environmental advantages. The situation caused travelers to experience a serious decrease in their enjoyment and appreciation of existing transportation, which resulted in a significant drop in their satisfaction levels. The existing transportation system’s rate of poor choices led both countries’ travelers to feel dissatisfied.

Based on the impacts on attitude, regret negatively influenced traveler attitudes in Malaysia and Thailand, supporting hypothesis 2. The study confirmed previous research^7,27^ that identified regret as having a significant influence on attitude. The study showed that regret had a marginally stronger influence on traveler attitudes in Malaysia when compared to Thailand. Travelers in these countries who experienced regret about their gas vehicle usage felt trapped in difficult situations. They became aware that gas vehicle emissions degraded air quality, leading to serious planetary damage. The current transportation usage led to negative self-perception and life outlook among travelers who felt guilty about their past consumption decisions. The persistent regret they experienced led to their pessimism regarding the importance of gas vehicles for consumers and their environmental impact on both society and nature. Second, satisfaction positively influenced traveler attitudes in Malaysia and Thailand, supporting hypothesis 4. The findings aligned with previous studies^30,31^ that demonstrated the substantial connection between satisfaction and attitude. The research indicated that satisfaction had a somewhat greater effect on traveler attitudes in Thailand compared to Malaysia. The travelers’ views showed changes in their appreciation for gas vehicles would result in either negative or positive reinforcement effects. The travelers noticed more challenges and disadvantages when they felt dissatisfaction with gas vehicles. The travelers showed more concern about environmental harm from carbon dioxide (CO2) emissions produced by gas vehicles. Transportation options became unfavorable for travelers once they developed resilient attitudes and mindsets.

Based on the impacts on discontinued intention, regret positively influenced discontinued intention in Malaysia and Thailand, supporting hypothesis 3. The study’s findings supported theoretical foundations^8,13^, demonstrating a strong connection between regret and discontinued intention. This research demonstrated that regret led to a higher discontinued intention among Thai travelers, while the effect remained slightly less pronounced among Malaysian travelers. The respondents’ views revealed that their regret demonstrated the adverse results stemming from their use of gas vehicles. The travelers experienced regret after realizing their use of current transportation methods had contributed to environmental changes and global warming. The circumstances led travelers to express emotional reactions about environmental sustainability values, which gas vehicles failed to meet. The travelers decided to stop using gas vehicles because their negative emotions motivated this choice. Second, satisfaction negatively influenced discontinued intention in Malaysia and Thailand, supporting hypothesis 5. The study’s results matched previous research^14,32^, which demonstrated the significant impact of satisfaction on customers’ intentions to discontinue usage. The study demonstrated that Malaysian travelers exhibited a stronger discontinued intention due to satisfaction compared to their Thai counterparts. As travelers lost their satisfaction with gas vehicles, they began to perceive these vehicles as having lower value and performance than their current expectations. Travelers shifted away from gas vehicles because they sought products that satisfied their personal and societal requirements as well as environmental benefits, which supported sustainable practices within their nations. The decreasing satisfaction with gas vehicles resulted in travelers obtaining fewer emotional rewards, which led them to stop using these vehicles for future travel. Finally, attitude negatively influenced discontinued intention in Malaysia and Thailand, supporting hypothesis, supporting hypothesis 6. The study findings supported the existing theory^15,33^, which identifies a major link between attitude and discontinued intention. The research confirmed the established theory but discovered that Thai travelers showed a stronger attitude effect on discontinued intention than Malaysian travelers. The outcome demonstrated how travelers perceive gas vehicles as having an effect on their environmental surroundings. The current situation revealed travelers expressing distrust and dissatisfaction concerning gas vehicles because they generate dangerous compounds, including carbon monoxide and nitrogen, that degrade air quality. Travelers identified gas vehicles that release carbon dioxide (CO₂) as analogous to greenhouse gases that drive global warming and climate change. The environmental downsides of gas vehicles caused travelers to identify them as inefficient transportation options that worsen the global environmental problems. The cultural focus on environmental stewardship in Thailand and Malaysia stands as a possible explanation. People in these countries already practice ethical consumption because of their cultural traditions that prioritize environmental sustainability. Understanding the environmental impact of gas vehicles helped them realize the importance of switching to eco-friendly vehicles. The second link connects to social pressures within these countries because environmental responsibility has become the standard, which means purchasing gas vehicles that damage their environment goes against social norms. The Thai and Malaysian travelers preferred to discontinue using gas vehicles because they wanted to avoid public criticism about their unsustainable product choices. Because of negative attitudes towards gas vehicles, travelers were likely to abandon traditional transportation methods and seek alternative options that promote sustainability for people, communities, and the natural world.

Based on the moderating impact, government policy significantly moderated the relationship between regret and discontinued intention in Malaysia and Thailand, supporting hypothesis 7. The findings validated existing theory^40,41^, which demonstrated government policy’s moderating effect between regret and discontinued intention. The study discovered that environmental protection policies that limit gas vehicle use reinforced the connection between personal regret and consumers’ intentions to stop using gas vehicles. The government policy required travelers to thoroughly comprehend each country’s environmental issues. The nationwide implementation of policy transformed traveler consumption behaviors into more sustainable practices, which helped lower each country’s carbon footprint. The traveler came to realize that gas vehicles fail to meet sustainable energy standards while observing the long-term negative consequences of their use, which led to their guilty feelings about environmental damage in their home country. The travelers developed a stronger wish to cease using gas vehicles in response to the rising sense of regret about their previous choices alongside the government policies in their respective countries. Travelers demonstrated greater interest in exploring and adopting alternative transportation solutions that employed renewable energy or produced fewer environmental pollutants such as carbon dioxide (CO2), nitrogen oxide, and carbon monoxide.

### Theoretical contributions

This study offers significant theoretical insights by creating and testing how traveler regret influences satisfaction and attitude, as well as the decision to stop using gas vehicles within this industry, while considering government policy effects. Regret functions as a psychological factor that affects satisfaction and attitude. Subsequently, satisfaction and attitude create travelers’ choices to abandon gas vehicles. Particularly, the individuals experience guilt, which leads to regret when they realize that their choice of gas vehicles as transportation methods results in environmental damage. The awareness of present issues generates dissatisfaction among travelers toward gas vehicles. The travelers develop a changed perspective regarding the value and environmental impact of gas vehicles following this realization. The persistent presence of doubt makes travelers lose faith in gas vehicles and recognize reduced benefits from their usage. The resulting alert informs all associated travelers, resulting in increased frustration and stress that leads to a strong intention to stop using gas vehicles.

Further, traveler satisfaction leads to negative attitudes and intentions to stop using gas vehicles. The contentment that comes from satisfaction creates positive feelings for travelers. Travelers who dislike gas vehicles develop negative feelings that lead them to form strong pessimistic views about those vehicles. Travelers lose confidence in gas vehicles, which prompts them to stop using them.

The third area of study reveals how people’s attitudes influence their decisions to stop using gas vehicles. Consumers who view gas vehicles negatively tend to perceive a reduced value in how these vehicles meet their functional requirements and expectations. Users are more knowledgeable about the environmental effects resulting from the use of current vehicles. These attitude outcomes lead to increased discontinued intentions that ultimately results in people switching behaviors in the future.

Finally, our analysis reveals how government policy moderates the connection between feelings of regret and customers’ intentions to discontinue gas vehicle usage. The strategy to cut down gas vehicle usage for environmental protection made travelers aware of these vehicles’ impact on their surroundings and Earth. Travelers begin to reassess their existing travel habits, which damage their environment, and realize the unsustainable nature of their practices. This creates feelings of regret about their use of gas-powered vehicles. As a result of their regret, they tend to stop using gas vehicles and choose alternatives that have less environmental impact.

### Practical implications

This study aims to aid policymakers, product engineers, and entrepreneurs in the automotive sector by elucidating consumer insights, specifically regarding traveler regret linked to gasoline-powered vehicles, a significant factor propelling the transition from traditional cars. In light of these findings, the subsequent customized techniques are recommended to assist the automotive sectors in Thailand and Malaysia in improving their sustainability initiatives.

Thai manufacturers should focus on improving fuel efficiency by creating compact and lightweight vehicles that meet the Energy Efficiency and Conservation Plan (EECP) requirements. The Thai government’s approval of ethanol-blended fuels like E20 and E85 should drive automakers to increase production of vehicles that run on these alternative fuel types. Bangkok’s congested urban traffic makes hybrid technology adoption especially beneficial because it helps lower fuel consumption. The Electric Vehicle Promotion Plan (EVPP) partnerships with the government enhance the transition by motivating manufacturers to produce electric and hybrid vehicles locally. Thai manufacturers must rapidly invest in electric vehicle manufacturing and charging infrastructure to meet the 30@30 strategy goals of producing 30% zero-emission vehicles by 2030 through partnerships in the Eastern Economic Corridor (EEC).

Malaysian automotive companies need to improve internal combustion engine efficiency according to the National Automotive Policy (NAP 2020), which places Energy Efficient Vehicles (EEVs) at the forefront. Considering Malaysia’s strong palm oil industry, manufacturers should assess increasing the use of biodiesel blends such as B10 and B20. Urban areas like Kuala Lumpur and Penang need incentives to promote hybrid technology because they face significant commuting distances and environmental challenges. Automotive manufacturers need to build local EV assembly facilities and battery factories as part of the government’s plan to develop a regional EV hub following the Low Carbon Mobility Blueprint guidelines. The nationwide growth of EV charging stations depends heavily on public-private partnerships, which guarantee infrastructure advances match the rising consumer needs.

By implementing these nation-specific solutions, manufacturers in Thailand and Malaysia may more adeptly address customer apprehensions over gasoline reliance and assume a proactive role in the shift towards sustainable mobility within the Southeast Asian framework.

## Conclusion

This study aimed to investigate how traveler regret influenced their satisfaction, attitude, and discontinued intention, along with government policy as the moderator. This study provided a statistical comparison between Malaysian and Thai travelers who experienced commuting through gas vehicles. Results revealed that regret significantly influenced traveler satisfaction in both countries. Meanwhile, regret and satisfaction also significantly influenced traveler attitudes in both countries. Furthermore, travelers’ discontinued intention in both countries was significantly influenced by regret, satisfaction, and attitude. Finally, the government policy of both countries moderated the relationship between regret and discontinued intention.

Traveler regret stands out as the primary factor shaping satisfaction levels and future intentions while also influencing attitudes within the gas vehicle industry, as shown by research from Malaysian and Thai travelers. Travelers understand the environmental damage caused by gas vehicles and feel remorseful about their contribution to environmental degradation. As a result, travelers show dissatisfaction and negativity toward existing transportation options. The government action toward pollution reduction increases traveler regret, which in turn strengthens their intention to abandon gas vehicle usage in Malaysia and Thailand. Travelers will likely abandon gas vehicle usage or choose alternative transportation powered by cleaner energy sources that support societal and environmental sustainability. The findings from this study conclude establish that gas-powered vehicles will inevitably become obsolete.

The main research objective is complete, yet certain limitations still exist. This study analyzed how various elements, including regret, satisfaction, attitude, and government policy, affect discontinued intention in gas vehicles, but other possible factors like anxiety, stress, complexity, and perceived value were not evaluated as being involved in the development of discontinued intention. Researchers studying this topic should include these key factors in their exploration processes for future studies. The data collection for this research utilized a convenience sampling approach. The data collection technique used in this study included potential bias because researchers might have unintentionally surveyed individuals from the target respondent group. Future research could address this concern by implementing cluster sampling methods to randomly choose full sample clusters. The research examined responses from respondents in Malaysia and Thailand, which means cultural and environmental contexts from both countries affected the results. Researchers in the future should try to extend this research model to perform cross-national comparative studies among different pairs of countries, such as China and South Korea or Indonesia and Laos, to examine various comparative situations.

## Electronic supplementary material

Below is the link to the electronic supplementary material.

Supplementary Material 1

## Data Availability

The datasets generated during the current study are not publicly available due to the need for strict protection of participants’ personal information.
