# Supplementary material for: Regret, policy, and discontinuance of gas vehicles: a cross-national study of Malaysia and Thailand
Source: Sci Rep. 2025 Jul 2;15:23020. doi: 10.1038/s41598-025-07259-0 (PMC12216853; doi:10.1038/s41598-025-07259-0)
Supplement: Supplementary file 1 — Supplementary Material 1 [file 41598_2025_7259_MOESM1_ESM.docx]

**Appendix**

| **Variable** | **Items** | **Pilot Testing (α)** | |
| --- | --- | --- | --- |
|  |  | **Malaysia** | **Thailand** |
| Regret | 1. I feel sorry to environment after using gas-vehicles.  2. I should have chosen non-gas vehicles for travelling options.  3. I feel sorry to human health after using gas-vehicles.  4. I really regret choosing gas-vehicles as my travelling options. | 0.920 | 0.816 |
| Satisfaction | 1. I think I am still happy to use the gas-vehicles.  2. Even travelling to nearby locations, I am still happy to go by gas-vehicles.  3. I think I am still satisfied with how well the functions of gas-vehicles operate.  4. I think I enjoy my travelling with the gas-vehicles, especially during holidays. | 0.811 | 0.783 |
| Attitude | 1. I think it is a good decision to use the gas-vehicles as a travelling option.  2. I think travelling with gas-vehicles can be a good option for other people too.  3. I think I still have a favorable opinion toward the gas-vehicle firms. | 0.779 | 0.901 |
| Government Policy | 1. I think government policy reducing gas-vehicle products is a good policy.  2. I think government policy reducing gas-vehicle products can help the country   to protect its environment.  3. I think government policy reducing gas-vehicle products can let many firms design new non-gas vehicles for future consumptions. | 0.806 | 0.882 |
| Discontinued Intention | 1. In the future, I will use gas-vehicles far less than today.  2. In the future, I will use another energy vehicles instead.  3. I will sometimes stop using the gas-vehicles for a while.  4. If possible, I will discontinue using the gas-vehicles now. | 0.761 | 0.718 |
